# Safety and Efficacy of Early Oral Feeding after Liver Transplantation with Roux-en-Y Choledochojejunostomy: A Single-Center Experience

**Published:** 2020

**Authors:** N. Fakhar, A. Sharifi, A. Chavoshi Khamneh, A. Kasraian Fard, Z. Heydar, S. H. Dashti, A. Jafarian

**Affiliations:** 1 *Liver Transplantation Research Center, Imam Khomeini Hospital Complex, Tehran University of Medical Sciences, Iran, Tehran*; 2 *Department of Surgery, Imam Reza Educational and Treatment Center, Tabriz University of Medical Sciences, Tabriz, Iran*; 3 *Department of General Surgery, Hamadan University of Medical Sciences, Hamadan, Iran*

**Keywords:** ERAS, Liver transplantation, Roux-en-Y Choledochojejenostomy

## Abstract

**Background:**

Early oral feeding, as one of the most important components of multimodal strategies referred to as Enhanced Recovery After Surgery (ERAS), is now widely adopted for optimization of post-operative recovery of surgical patients.

**Objective:**

To assess ERAS outcome in patients who underwent liver transplantation in our center.

**Methods:**

In a prospective study, patients who underwent liver transplantation from April 2015 to June 2018 at Imam Khomeini Hospital Complex, affiliated to Tehran University of Medical Sciences, Tehran, Iran, were enrolled in this study. Serum albumin, total iron-binding capacity (TIBC), and course of hospital stay were assessed.

**Results:**

39 (23 male) patients who underwent choledochojejunostomy with Roux-en-Y anastomosis for liver transplantation were enrolled. The mean±SD pre-operative serum albumin and TIBC levels of patients were 3.0±0.6 (range: 1.9–4.1) g/dL and 304±75 (range: 154.0–437.0) µg/dL, respectively. The mean±SD time between the end of operation and starting oral feeding was 11.6±1.8 (range: 9.0–15.0) hours. All patients tolerated early oral feeding with liquids followed by solid foods; no vomiting reported in patients. Overall, patient survival rates at one month and three months were 89.7% and 89.7%, respectively. In our study, no leak of anastomosis was reported.

**Conclusion:**

There was no major harm for ERAS after liver transplantation and it might be even helpful as in colorectal surgeries. As seen in our study, oral feeding was started as soon as possible after the end of operation in almost all patients and all of them tolerated early oral feeding. No one had vomiting or nausea.

## INTRODUCTION

Unlike some traditional standards in surgical care, it has been recently documented that starvation during post-operative period after a major surgery is not necessary and may even be harmful [1]. Early oral feeding, as one the most important components of multimodal strategies referred to as Enhanced Recovery After Surgery (ERAS), is now widely adopted for optimization of post-operative recovery of surgical patients [2].

ERAS programs were developed in colorectal surgeries; later on, they were successfully implemented in other major surgeries including gastrectomy and abdominal hysterectomy and resulted in lower morbidity rates and shorter hospital stay [3-6]. Recently, a few studies are available about the safety and effectiveness of ERAS in liver resection and pancreaticoduodenectomy surgeries reporting inconsistent results regarding post-operative complications and hospital stay [7-9]. However, to the best of our knowledge, there is no study to evaluate the outcomes of early oral feeding in patients undergoing liver transplantation with Roux-en-Y choledochojejunostomy.

We therefore conducted this study to assess the safety and feasibility of early oral feeding in patients undergone liver transplantation with choledochojejunostomy with Roux-en-Y anastomosis.

## PATIENTS AND METHODS

In a prospective study, patients who underwent liver transplantation from April 2015 to June 2018 at Imam Khomeini Hospital Complex, affiliated to Tehran University of Medical Sciences, Tehran, Iran, were enrolled in this study. The included patients were recipients who underwent choledochojejunostomy with an Roux-en-Y anastomosis during liver transplantation. Patients who were not conscious early after the surgery or died intra-operatively were excluded from the study. The study was conducted after obtaining the approval from the Ethics Committee of our hospital and Tehran University of Medical Sciences. The necessary information was provided to patients a day before the operation. An informed written consent was obtained from each patient.

A 5-mL blood sample was taken from each patient just before the operation to check for serum albumin and total iron-binding capacity (TIBC). Habitual substance use including cigarette, opioid and alcohol was recorded subjectively as “never;” “former user,” defined as abstinence for at least 6 months; and “current user.” All liver transplantation surgeries were performed by the same team of surgeons. Temporary portocaval shunt or venovenous bypass was not used in any patients. Biliary anastomosis was done between the end of the common bile duct of the donor’s liver and side of the 40-cm Roux limb of the jejunum of the recipient. A nasogastric tube was passed into the stomach at the end of the operation. All patients were transferred to the intensive care unit immediately after the operation and visited frequently by the same surgeon. Post-operatively, nasogastric tube was pulled out within one hour after extubation and at the time the patient became fully conscious. Then, soft liquid diet was started; in absence of vomiting, it was advanced to normal food after one day. Otherwise, oral feeding was stopped until nausea was resolved. Pre-operative data including demographics, laboratory data, and habitual status, inrta- and post-operative data were recorded prospectively.

Statistical Analysis

Data analysis was performed using SPSS^®^ for Windows^®^ ver 22.0 (SPSS Inc, Chicago, IL, USA). Qualitative and quantitative data were compared using Fisher’s exact, and Kruskal-Wallis tests, respectively. Patient survival rates were computed with Kaplan-Meier method at 1 month and 3 months after liver transplantation. A p value <0.05 was considered statistically significant.

## RESULTS

During the study period, 404 patients underwent liver transplantation from cadaveric donors. Thirty-nine (23 male) patients who underwent choledochojejunostomy with Roux-en-Y anastomosis for liver transplantation were enrolled in this study.

The mean±SD age and body mass index (BMI) of patients were 37.0±15.4 (range: 1–61) years, and 22.0±3.7 (range: 16–30) kg/m^2^, respectively. The mean±SD MELD score of patients was 20.3±6.3 (range: 8–37); eight had a MELD score ≥28.

The etiology of end-stage liver disease and comorbidities of patients are listed in Table 1. Information regarding habitual substance use in patients is presented in Table 2.

**Table 1:** The frequency distribution of etiologies of end-stage liver disease and comorbidities in our patients

Etiology	n (%)
Primary sclerosing cholangitis	23 (59)
Re-transplantation	9 (23)
Acute liver failure	2 (5)
Non-alcoholic steatohepatitis	2 (5)
Others	3 (8)
Comorbid disease	
Inflammatory bowel disease	8 (20.5)
Diabetes mellitus type 2	8 (20.5)
Hypothyroidism	2 (5)

**Table 2:** Characteristics of habitual substance use in patients

Substance	Never	Former	Current
Cigarette smoking	34	4	1
Opioid use	36	3	0
Alcohol drink	38	1	0

The mean±SD pre-operative serum albumin and TIBC levels of patients were 3.1±0.6 (range: 1.9–4.1) g/dL, and 300±77 (range: 154–437) µg/dL, respectively. There was no significant (p=0.35) difference in pre-operative level of serum albumin between the patients who underwent Roux-en-Y anastomosis (3.1±0.6 g/dL) and those who underwent duct-to-duct anastomosis (3.2±0.6 g/dL). Nor does it for pre-operative TIBC—300±77 µg/dL in Roux-en-Y patients and 278±88 µg/dL in duct-to-duct patients (p=0.30).

The mean±SD pre-operative serum albumin in Roux-en-Y patients with the MELD score <28 was 3.2±0.5 g/dL, higher than that in those with a score ≥28 (2.5±0.4 g/dL). The mean±SD operative time was 292±52 (range: 191–404) minutes.

Early oral feeding was started as planned in all patients post-operatively one hour after extubation, with soft liquid diet, except for one patient who had primary non-function after liver transplantation. All of our operations were started at the evening and were finished at night; so for patients’ safety, we extubated patients early in the morning after the patients retained full consciousness. One hour after extubation, we allowed the patients to start soft liquid diet. The mean±SD time between the end of operation and starting oral feeding was 11.6±1.8 (range: 9.0–15.0) hours. All patients tolerated early oral feeding with liquids followed by solid foods; no vomiting was reported. If patients had nausea, we used one dose of intravenous metoclopramide and hold the diet for an hour; then, the patient was allowed to starting the diet again.

Overall, the patient survival rates at one month and three months were 89.7%, and 89.7%, respectively. The cause of death was sepsis in two patients at post-operative days 10 and 16, primary non-function in one patient at post-operative day three, and disseminated intravascular coagulation in another patient at post-operative day 30. In our study, no leak of anastomosis was reported.

In alive patients, the mean±SD hospitalization days was 21.0±14.0 days in patients with Roux-en-Y anastomosis and 14.1±7.2 days in duct-to-duct patients (p<0.0001) (Table 3).

**Table 3:** Frequency distribution of patients according to their hospital stay after transplantation and treatment groups

Group	Hospitalization days				Total
	≤10	11–20	21–30	>30	
Roux-en-Y	11 (28%)	13 (33%)	6 (15%)	9 (23%)	39 (100%)
Duct-to-duct	146 (40.0%)	145 (39.7%)	33 (9.0%)	41 (11.2%)	365 (100%)

In patients who were discharged within the first 10 days of operation, there was no significant (p=0.83) difference between Roux-en-Y and duct-to-duct groups during hospitalization (8.5±1.4 vs. 8.4±1.2 days, respectively). In patients who admitted for ≥20 days after the operation, there was no significant difference in this regard (13.0±4.2 days in Roux-en-Y patients vs. 11.7±3.7 days in duct-to-duct group, p=0.13).

However, when we compared hospitalization duration in patients who admitted >20 days, Roux-en-Y patients had more duration of hospitalization than duct-to-duct group (16.3±7.4 days vs. 13.1±5.4 days, respectively, p=0.006).

## DISCUSSION

ERAS was first used in colorectal surgery. Few studies are available regarding its efficacy in hepatobiliary operations [10, 11]. However, many basic concepts of ERAS in hepatobiliary surgery have derived from colorectal field. Therefore, there might be some misleading points. For example, hepatobiliary surgeries often take a longer time than colorectal operations, and pre-op fluid therapy is quite different. Anastomosis site is more proximal. In liver surgery, to lessen intra-operative blood loss, central venous pressure is rather to be low and less intravenous fluid is given to patient pre-operatively. But, in colorectal surgeries, especially those with an ostomy, replacement of excretions is essential. These differences take under question the generalizability of ERAS principles to hepatobiliary surgery. Therefore, performing some investigations in this filed is necessary [12]. ERAS is not evolving as a new strategy in many fields like cesarean section [13, 14], pediatric operations [15], and spine surgery [16].

We used a fast-track method for patients after liver transplantation with Roux-en-Y anastomosis to assess post-operative consequences. Our goal was to design a system to create a better post-operative condition for patients, decrease their hospital stay and complications together with educating them essential principles to have a near normal life after discharge. As seen in our study, oral feeding was started as soon as possible after the end of the operation in almost all patients; they all tolerated early oral feeding. No one had vomiting. However, the overall survival rates were 89.7% and 89.7% one month and three months post-operative, respectively. Patients passed an almost uneventful post-operative period.

Length of stay (LOS) in hospital is of paramount importance. As many complications like hospital-acquired infection is closely associated with LOS. Also, it determines hospital expenses for each patient and patients’ turn over [17]. But, trying to decrease the LOS is not only associated with the medical team but also linked with patients’ education before the operation including his or her expectations, pre-operative training regarding post-operative cooperation with the medical team for faster mobilization or coughing enough to prevent atelectasis. Many studies have investigated LOS. For instance, 161 patients undergoing elective liver resection were assessed. They reported that the median LOS was reduced to 6 days using ERAS [18].

MacKay, *et al*., in 2008 assessed early discharge following liver resection for colorectal metastases and reported that the median LOS was 4 days [19]. Nonetheless, LOS per se does not indicate safety of ERAS. A Cochrane review in colorectal surgery concluded that post-operative complications would be a better indicator of assessment [20].

Another cross-sectional study performed in 2011 by De-Xin Lin, assessed 117 patients who underwent elective liver resection. They confirmed that a fast-track clinical strategy in their patients could enhance hepatic surgical procedures by monitoring and improving quality of care. They found that decreasing post-operative ICU stay, early patient ambulation and imitation of oral feeding as soon as possible, lessening intravenous fluid to a minimum level, and early removal of nasogastric tubes, urinary catheters or intra-abdominal drain decrease the post-operative LOS [21].

In our study, we observed that in patients with <20 days of hospital stay, there was not significant difference in Roux-en-Y patients with early oral feeding compared with other patients in terms of duration of hospitalization. In patients with hospital stay of >3 weeks, Roux-en-Y group with early oral feeding had longer hospitalization than others; however, these finding may be attributed to other complications such as sepsis.

A systematic review of 11 articles published between 2007 and 2011 shows the safety and feasibility of ERP in hepatopancreaticobiliary (HPB) surgery. Many basic concepts of ERAS in HBP surgeries have been borrowed from the colorectal ERP [12].

One of the main concerns regarding ERAS in HPB surgeries is anastomosis leak of pancreas when early feeding is initiated. However, published articles regarding fast-track feeding have not shown more complications such as anastomosis leak [16, 17, 23, 24]. Although, many surgeons believe in bowel rest after surgery, with a justification that it might lessen the probability of anastomotic leak, it was not a matter of concern in our investigation, as we did not have anastomosis on pancreas; it is an important issue for operations like Whipple surgery.

Our study had some limitations. First, the study design was observational and had a cross-sectional identity. Therefore, comparing with other groups of patients who experienced bowel rest after surgery was not possible. We should compare the Roux-en-Y patients in two randomized groups with and without ERAS protocol to reaching more reliable results. Also, as hepatic implantation is considered an advanced operation, performing a multi-center study with more patients operated with different teams of surgery may uncover the real outcome. The very low complications rate in our study might be due to our experience in a high-volume center of transplantation.

In conclusion, by considering the above-mentioned shortages, it is thought that there is no major harm for ERAS after liver transplantation with Roux-en-Y chledochojejunostomy and it might be even helpful as it is in colorectal surgeries.
